# Evaluation of the diagnostic indices and clinical utility of qualitative cardiodetect^® ^test kit in diagnosis of ami within 12 hours of onset of chest pain in the emergency department

**DOI:** 10.1186/1865-1380-4-67

**Published:** 2011-10-27

**Authors:** Nik Hisamuddin NAR, Ahmad Suhailan M

**Affiliations:** 1 School of Medical Sciences, USM, Kubang Kerian, Malaysia; 2 Specialist Emergency Medicine, Hospital Kuala Lumpur, Malaysia

## Abstract

**Introduction:**

Cardiac biomarkers may be invaluable in establishing the diagnosis of acute myocardial infarction (AMI) in the ED setting.

**Objective:**

To assess the diagnostic indices of the Cardio Detect assay and the quantitative cardiac troponin T test, in diagnosing AMI in the ED, according to the time of onset of chest pain.

**Methodology:**

A total of 80 eligible patients presenting with ischemic type chest pain with duration of symptoms within the last 36 h were enrolled. All patients were tested for H-FABP and troponin T at presentation to the ED. A repeated Cardio Detect test was performed 1 h after the initial negative result, and a repeated troponin T test was also performed 8-12 h after an initial negative result. The diagnostic indices [sensitivity, specificity, positive predictive value, negative predictive value, receiver operating curve (ROC)] were analyzed for Cardio Detect and Troponin T (individually and in combination) and also for the repeat Cardio Detect test. Data entry and analysis were performed using SPSS version 12.0 and Analyze-it software.

**Results:**

The Cardio Detect test was more sensitive and had a higher NPV than the troponin T (TnT) test during the first 12 h of onset of chest pain. The repeat Cardio Detect had better sensitivity and NPV than the initial Cardio Detect. The sensitivity and NPV of the combination test (Cardio Detect and troponin T) were also superior to each test performed individually.

**Conclusion:**

The Cardio Detect test is more sensitive and has a better NPV than the troponin T test during the first 12 h of AMI. It may be used to rule out myocardial infarction during the early phase of ischemic chest pain.

## Background

Early and correct diagnosis of patients admitted to the hospital with symptoms suggestive of acute myocardial infarction (AMI) is paramount to ensure appropriate therapy is given to minimize myocardial injury and improve clinical outcome [1]. The urgency in recognizing and treating patients with an AMI as early as possible has been repeatedly stressed and reiterated in various guidelines that lead to the well-known phrase of 'time loss is myocardium loss'. With the passing of time and further delay in diagnosing AMI and administration of reperfusion therapy, more cardiac muscle will be damaged [2]. As a consequence, the patient's prognosis will deteriorate. To expedite the diagnosis, the AHA (American Heart Association) Guidelines for the management of patients with ST-elevation myocardial infarction (STEMI) in 2004 recommended that an electrocardiogram (ECG) should be performed and interpreted by an experienced physician within 10 min of arrival to the emergency department (ED). If reperfusion therapy is deemed indicated, the decision whether to use fibrinolytic therapy or percutaneous coronary intervention (PCI) should be made within the next 10 min [3,4].

It is equally important to identify patients who are not suffering from AMI and who can be sent home safely early after admission. This will avoid unnecessary inpatient hospital admission, which is usually accompanied by numerous invasive tests. The diagnosis of AMI has to be accurate and precise. In the unfortunate event that a patient is mistakenly treated for AMI without having the condition, unwarranted risk and complications may result from the reperfusion therapy. This not only poses an imminent danger to the patient, but also is a potential source of litigation. Another scenario with potentially catastrophic consequences is discharging a patient who is actually suffering from a myocardial infarct. About 2-8% of patients who presented with chest pains to the ED were misdiagnosed and sent home [5]. The morbidity and mortality in these patients were high. Patients could be misdiagnosed and inappropriately discharged especially if they presented with atypical chest pain and non-diagnostic ECG changes [6].

Therefore, diagnosing an AMI as early and as accurately as possible is the most critical phase in the treatment of a patient presenting with chest pain to the ED. Once a definitive diagnosis can be made, prompt steps can be taken to limit the myocardial necrosis, including instituting reperfusion therapy. The World Health Organization (WHO) criteria for the definition of AMI includes a combination of two out of three characteristics composed of clinical history, a rise and fall of cardiac biomarkers, and ECG changes. Despite being guided by the WHO criteria, the diagnosis of AMI may still be challenging in many instances. Patients may present with atypical symptoms, or myocardial necrosis may occur without any symptoms at all [7,8]. Not all patients who develop myocardial necrosis exhibit ECG changes. Approximately 40% of patients with AMI showed no diagnostic ECG changes on admission. It has been reported that 50% of the AMI patients who were admitted with acute chest pain did not have any diagnostic changes on initial ECG tracing. Therefore, a normal ECG does not rule out the diagnosis of MI [9-11].

In situation like these, cardiac biomarkers may be invaluable in establishing a diagnosis of AMI in the ED setting. A number of established cardiac biomarkers have been available on the market, and several new promising assays with better sensitivity have been discovered. In April 2000, the Joint European Society of Cardiology/American College of Cardiology Committee (ESC/ACC) for the Redefinition of Myocardial Infarction published new criteria for the diagnosis of AMI. They proposed the use of cardiac troponin (I or T) as the most sensitive and specific marker of AMI. This revised definition of AMI has reiterated the importance of cardiac-specific markers of necrosis, specifically the cardiac troponins, as crucial determinants for the diagnosis of AMI [12].

A recent potential cardiac biomarker that shows release kinetics similar to myoglobin is heart-type fatty acid-binding protein (H-FABP). It is a low-molecular-weight cytoplasmic protein (15 kDa) that is present in abundance in the cytosol of cardiac myocytes [13,14]. It is undetected in normal conditions, but is rapidly released into the circulation after myocardial cell damage. Many studies have been conducted on H-FABP, but few have investigated the diagnostic accuracy and practicality of Cardio Detect^®^. We believe that this diagnostic kit, which detects H-FABP at the bedside, still needs further evaluation, especially to assess its performance and practicality to detect AMI in patients presenting with chest pain in the ED setting.

### General objectives

The diagnostic indices and clinical utility of qualitative Cardio Detect^® ^test kit in the diagnosis of AMI in the emergency department was evaluated in comparison to the quantitative cardiac troponin T assay.

### Specific objectives

1. To compare the diagnostic indices [sensitivity, specificity, positive predictive value, negative predictive value, receiver operating characteristic (ROC) curve] of the qualitative Cardio Detect^® ^assay and the quantitative cardiac troponin T test in diagnosing AMI in the ED according to the time of onset of chest pain.

2. To verify whether there was any improvement in the diagnostic indices (sensitivity, specificity, positive predictive value, negative predictive value, ROC) of the Cardio Detect^® ^test in diagnosing AMI when repeated 1 h after an initial negative result in patients with acute ischemic type chest pain presenting to the ED.

3. To determine whether there was any improvement in the diagnostic indices (sensitivity, specificity, positive predictive value, negative predictive value, ROC curve) of the Cardio Detect^® ^assay in diagnosing AMI when used in combination with the cardiac troponin T test in patients with acute ischemic chest pain presenting to the ED.

## Methodology

This study was a prospective cross-sectional study. It was conducted from February 2008 until September 2008, and the source population was all patients who presented with chest pain suggestive of AMI to a regional tertiary center with an attendance rate exceeding 70,000 patients per year. Ethical approval was obtained from the department board review and hospital ethics committee on 13 February 2008 [reference USMKK/PPP/JEPeM 199.3(10)]. A short-term grant was approved by the School of Medical Sciences, USM. The eligible population was the source population fulfilling the inclusion and exclusion criteria.

### Inclusion criteria

1. Adult patients 18 years old or above.

2. All patients presenting to the ED with ischemic chest pain that was less than 36 h duration of onset.

### Exclusion criteria

1. Patients with a history of recent muscle injury (< 3 days), including intramuscular injection.

2. Patients with acute or chronic skeletal muscle damage or disorders including rhabdomyolysis, dermatomyositis, muscular dystrophy, and polymyositis.

3. Patients with renal insufficiency as defined by serum creatinine > 200 μmol/l.

4. Critically ill patients, including those with cardiogenic shock, septic, intubated and ventilated patients.

5. Patients who had had a recent myocardial infarction or received fibrinolytic therapy or angioplasty within the last 14 days prior to presentation to the ED.

The sampling method for this study was obtained through convenience sampling. Patients were enrolled during all shifts and days of the week. The sample size was calculated by a biomedical statistician with reference to 'Statistical Evaluation of medical tests for classification and prediction; study design and hypothesis testing' (Margaret Sullivan Pepe, Oxford University Press 2003). The variables used in the calculation were as follows:

Type I error is 5% (α = 0.05)

Power of study = 0.8

Eighty-seven patients required, which included a 20% dropout rate in this study.

**Figure 1:**
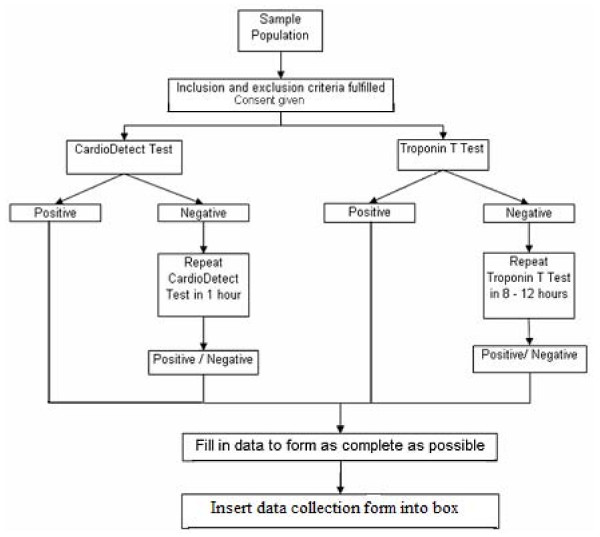
**Flow chart of the study**.

**Figure 2:**
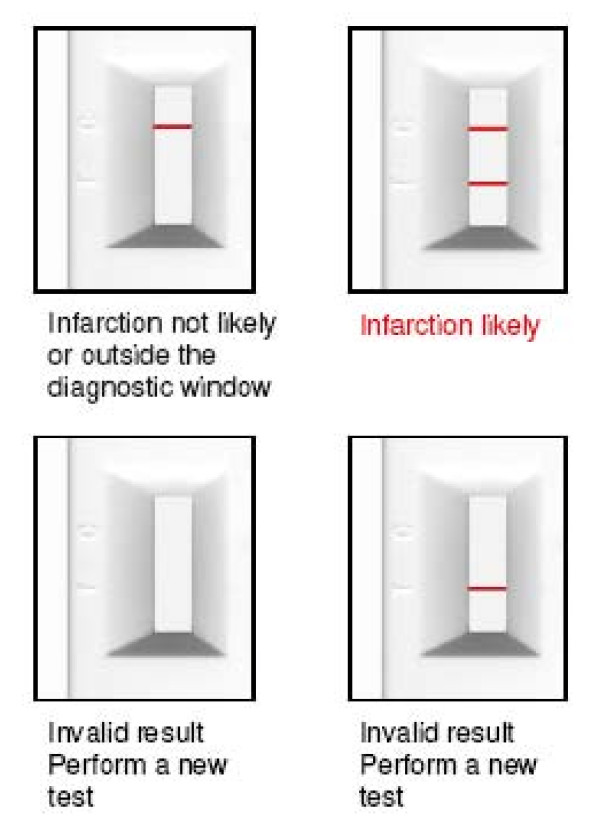
**Visual interpretation of Cardio Detect med card**.

Upon arrival at the emergency department (ED), all patients with chest pain suggestive of myocardial infarction were triaged. These cases were fast tracked, and seen by a paramedic or medical officer as soon as possible. An ECG was performed mostly within 10 min of presentation to the ED. The ECG was repeated after 1 h of the first ECG if indicated. After informed consent was obtained, a blood sample was drawn either through a needle or aspirated via an intravenous cannula; 10 ml of blood was drawn into a plastic syringe without added heparin. A portion of the blood was tested for both TnT and H-FABP, irrespective of the ECG findings. The remaining blood samples were tested for other routine blood investigations, including full blood count (FBC), renal profile (RP), and cardiac enzymes (creatine kinase). Presence of H-FABP in the circulation was detected using the point-of-care Cardio Detect^® ^med card. The med card was stored in a designated refridgerator in the ED Satellite Laboratory. All med cards were sealed in a plastic pouch and kept between the temperatures of 2-8°C, as recommended by the manufacturer (rennesens GmbH: instructions for use). As the med card is retrieved, the plastic pouch is opened, and it is placed horizontally on an even surface (Figures 1 and 2).

The cardiac troponin T (TnT) test was performed using the Cardiac Reader analyzer (Roche Diagnostics) located in the satellite laboratory in the ED. It is a qualitative assay of TnT in heparinized venous blood. A portion of the blood sample drawn from the patient was inserted into a heparinized tube provided with the TnT test kit. Regardless of patient's decision to participate in the study or not, all received routine institutional care. Treatment for AMI was not withheld. Based on predetermined criteria, the attending physician (emergency physician or medical physician) made the final diagnosis, and subjects were classified into two groups: (1) acute myocardial infarction and (2) non-acute myocardial infarction. The diagnosis of AMI is made based on the redefinition of AMI by ESC/ACC and/or the WHO criteria.

Data entry and analysis were performed with Statistical Packages for Social Sciences (SPSS version 11.0 for Windows, Chicago, IL), which were licensed to the School of Medical Sciences, University Sains Malaysia. Mean and standard deviation were obtained for all the numerical variables (age and serum creatinine). Descriptive statistics (frequencies) were obtained for all patients such as age, gender, comorbidity, and past medical history. The independent variables were all patients presenting with chest pain (categorical) as in the inclusion criteria, including the timing of onset (numerical) of chest pain. The dependent variables included the qualitative (positive or negative) outcome of the bedside test for both test kits. Sensitivity, specificity, PPV, NPV, and ROC were obtained for the Cardio Detect and TnT (individually and in combination) and for the repeated Cardio Detect test. All diagnostic indices were determined for each test under consideration at the following interval from the onset of chest pain: (1) 4 h or less (group 1), (2) more than 4 h but 12 h or less (group 2), (3) more than 12 h but 24 h or less (group 3), and (4) more than 24 h after onset of chest pain (group 4).

## Results

Eighty patients were recruited into the study, of which 62 (77.5%) were male and 18 (22.5%) were female. The recruitment number was still within the required sampling of 20% dropout (minimum of 70 patients needed).

**Figure 3:**
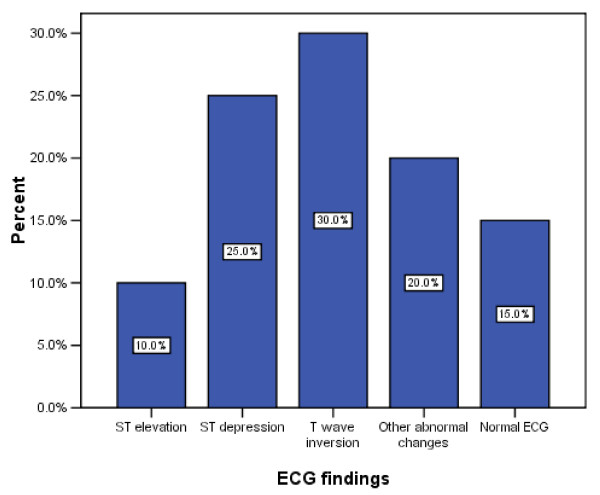
**The ECG findings recorded at presentation to the ED**.

Baseline characteristics for the study population are shown in Table 1. Thirty-two patients (40%) turned up at the ED within 4 h after onset of chest pain (group 1 = 32 patients). Thirty-one patients (38.8%) presented after 4 h, but within 12 h of chest pain (group 2 = 31 patients). Thirteen (16.3%) subjects came to the ED after 12 h but within 24 h of chest discomfort (group 3 = 13 patients). Only four patients (5%) presented late to ED after more than 24 h of onset of chest pain. Figure 3 shows the initial ECG findings in the ED. The majority of patients (72.5%) in this study did not have an AMI. Twenty-two patients (27.5%) were diagnosed with AMI as the final diagnosis made by the attending physicians. Of the 22 patients diagnosed with AMI, 14 (63.6%) had non-ST elevation myocardial infarction (NSTEMI). The remaining eight patients (36.4%) had ST elevation myocardial infarction (STEMI). Out of the 32 patients who presented to the ED within 4 h after the onset of chest pain, 10 (31.2%) had AMI (group 1). Six of the 31 patients (19.3%) who presented after 4 h but within 12 h of chest pain had AMI (group 2). Five of the 13 patients (38.4%) had AMI in the group of patients who turned up after 12 h but within 24 h of chest pain (group 3). Finally, one of the four (25%) late presenters who came after 24 h of chest pain had AMI (group 4).

**Table 1:** Baseline characteristics of the study population.

Characteristics	Study population	AMI	No AMI
**N**	80	22	58
**Age (years)** **(mean ± SD)**	58.96 ± 12.4	59.45 ± 13.9	58.78 ± 11.9
**Men/women**	62/18	20/2	42/16
**Diabetes**	22	6	16
**Hypertension**	43	10	33
**Hyperlipidemia**	26	6	20
**Smoking**	38	13	25
**Previous history of** **CVD**	44	9	35
**Serum creatinine** **(μmol/l ± SD)**	115.1 ± 28.5	117.0 ± 27.4	114.4 ± 29.1
**Family history of** **heart disease**	22	5	17

Tables 2, 3, and 4 summarize the diagnostic indices for Cardio Detect, repeated Cardio Detect, TnT, and combination tests.

**Table 2:** Diagnostic indices of Cardio Detect and TnT.

Parameter	Cardio Detect (95% CI)	TnT (95% CI)
**Sensitivity (%)**		
≤ 4 h	50.0 (20.1-79.8)	10.0 (0.5-45.9)
> 4 but ≤ 12 h	83.3 (42.0-99.2)	66.6 (30.2-94.8)
> 12 but ≤ 24 h	100.0 (46.2-100.0)	100.0 (46.3-100.0)
> 24 h	0.0 (0.0-94.5)	100.0 (5.4-100.0)
**Specificity (%)**		
≤ 4 h	63.6 (40.8-81.9)	100.0 (81.5-100.0)
> 4 but ≤ 12 h	52.0 (33.7-72.8)	100.0 (83.9-100.0)
> 12 but ≤ 24 h	25.0 (3.9-59.8)	100.0 (62.8-100.0)
> 24 h	66.6 (12.5-98.2)	100.0 (30.9-100.0)
**PPV (%)**		
≤ 4 h	38.4 (15.1-67.7)	100.0 (5.4-100.0)
> 4 but ≤ 12 h	29.4 (14.3-58.8)	100.0 (46.2-100.0)
> 12 but ≤ 24 h	45.5 (16.4-71.4)	100.0 (46.2-100.0)
> 24 h	0.0 (0.0-94.5)	100.0 (5.4-100.0)
**NPV (%)**		
≤ 4 h	73.6 (48.5-89.8)	70.9 (51.7-85.1)
> 4 but ≤ 12 h	92.9 (66.0-99.6)	92.5 (75.0-98.7)
> 12 but ≤ 24 h	100.0 (19.7-100.0)	100.0 (62.8-100.0)
> 24 h	66.6 (12.5-98.2)	100.0 (30.9-100.0)

**Table 3:** Diagnostic indices for repeat Cardio Detect and combination tests.

Parameter	Repeat Cardio Detect (95% CI)	Cardio Detect and TnT (combination test)
**Sensitivity (%)**		
≤ 4 h	60.0 (17.0-92.7)	60.0 (27.3-86.3)
> 4 but ≤ 12 h	50.0 (2.60-97.3)	100.0 (56.0-100.0)
> 12 but ≤ 24 h	-	100.0 (46.2-100.0)
> 24 h	-	100.0 (5.4-100.0)
**Specificity (%)**		
≤ 4 h	85.7 (58.3-97.6)	63.6 (40.8-81.9)
> 4 but ≤ 12 h	50.0 (31.6-68.3)	52.0 (33.7-72.8)
> 12 but ≤ 24 h	-	25.0 (3.9-59.8)
> 24 h	-	66.6 (12.5-98.2)
**PPV (%)**		
≤ 4 h	60.0 (17.0-92.7)	42.8 (18.8-70.3)
> 4 but ≤ 12 h	14.2 (0.3-32.2)	33.3 (17.2-61.3)
> 12 but ≤ 24 h	-	45.5 (16.4-71.4)
> 24 h	-	50.0 (2.6-97.3)
**NPV (%)**		
≤ 4 h	85.7 (58.3-97.6)	77.7 (51.9-92.6)
> 4 but ≤ 12 h	85.7 (67.7-99.6)	100 (73.2-100.0)
> 12 but ≤ 24 h	-	100.0 (19.7-100.0)
> 24 h	-	100 (19.7-100)

**Table 4:** Area under the ROC curves (AUC).

Test	Area	P-value	95% CI
**≤ 4 h (group 1)**			
Cardio Detect (CardioD)	0.568	0.542	0.350-0.787
Troponin T (TropT)	0.550	0.655	0.325-0.775
Repeated CardioD	0.733	0.127	0.449-1.017
Combine CardioD & TropT	0.744	0.049	0.520-0.969

**> 4 but ≤ 12 h (group 2)**			
CardioD	0.698	0.113	0.493-0.903
TropT	0.857	0.004	0.650-1.064
Repeated CardioD	0.500	1.000	-0.102 to 1.102
Combine CardioD & TropT	0.769	0.031	0.608-0.930

**> 12 but ≤ 24 h (group 3)**			
CardioD	0.611	0.505	0.308-0.914
TropT	1.000	0.003	1.000-1.000
Combine CardioD & TropT	0.611	0.505	0.308-0.914

**> 24 h (group 4)**			
CardioD	0.333	0.655	-0.283 to 0.949
TropT	1.000	0.180	1.000 - 1.000
Combine CardioD & TropT	0.833	0.371	0.384-1.282

*The closer the ROC curve comes to the 45-degree diagonal line of the ROC space, the less accurate the test. An AUC of 1 represents a perfect test; elsewhere an area of 0.5 represents an uninformative test.

## Discussion

Myocardial infarction reflects the cell death of cardiac myocytes caused by prolonged ischemia, which is the result of a perfusion imbalance between supply and demand. It occurs when myocardial ischemia exceeds a critical threshold and overwhelms myocardial cellular repair mechanisms that are designed to maintain normal operating function and homeostasis [15]. If the resultant ischemia is severe enough to cause sufficient myocardial damage, detectable quantities of cardiac biomarkers will be released into the bloodstream [16]. Cardiac biomarkers have characteristic release and clearance kinetics. Thus, the time to presentation and comorbidities that affect clearance may confound the interpretation of biomarkers [17]. They are released from necrotizing myocardium in a time-specific manner. Biochemical markers of myocardial injury, such as cardiac troponin and creatine kinase (CK), are detected in plasma approximately 4-6 h after the onset of myocardial injury, and their plasma level returns to normal after 7-10 days for cardiac troponin and 50-70 h for CK [18]. Myoglobin is the earliest biochemical marker of myocardial cell damage, and it is detectable in blood within 1 to 2 h of myocardial damage [19].

Serial sampling of multiple cardiac markers beginning at the time of presentation is currently recommended [20]. The sensitivity of serial measurements of multiple markers nears 100%, whereas the sensitivity of a single measurement of any biomarker at the time of presentation is poor. The recommended time between the first and second blood draw is 6 to 7 h [21,22]. If cardiac marker levels are not elevated but clinical suspicion remains high, a third set of markers should be drawn at 12 to 24 h after presentation [23]. A multimarker approach with different release kinetics to diagnose AMI was also recommended. H-FABP is a low-molecular-weight cytoplasmic protein (15 kDa) that is present in abundance in the cytosol of cardiac myocytes. It plays an important role in the uptake and oxygenation of long-chain fatty acids in the heart [24,25]. It is undetected in normal conditions, but is rapidly released into the circulation after myocardial cell damage. Plasma level rises as early as 1-3 h after AMI. H-FABP level peaks at 6-8 h and returns to normal within 24-36 h after the initial insult [26-29].

The Cardio Detect^® ^was more sensitive and had a higher NPV than TnT during the first 12 h of onset of chest pain. The higher sensitivities of Cardio Detect^® ^in the early phase of chest pain were also reported in other studies [30,31]. The sensitivity of Cardio Detect^® ^in group 1 (≤ 4 h) was 50% and in group 2 (> 4 h but ≤ 12 h) was 83.3%, compared with 10% and 71.4% of TnT. This could be explained by the fact that H-FABP is released into the circulation as early as 30 min after myocardial necrosis and reaches a peak level at 7 to 9 h. Therefore, H-FABP can be detected earlier in the circulation after the onset of AMI. In contrast, TnT starts to rise to greater than threshold values 3-6 h after the onset of AMI and reaches a peak after 14 to 18 h. The sensitivity of TnT was expected to be low during the early phase of chest pain, since cardiac troponin may not be detectable for up to 6 h after the onset of chest pain. The sensitivity of Cardio Detect and TnT improved over time and reached 100% in patients from group 3. The sensitivity of Cardio Detect decreased after 24 h of chest pain (group 4). This is because the H-FABP level normalizes in the circulation after 24 h, hence explaining the drop in sensitivity. The ROC curve is a useful graphic method for comparing different tests [32]. Comparison of the ROC curves of Cardio Detect^® ^and TnT (Table 4) in patients from group 1 showed that no test was apparently superior to the other. The AUC for Cardio Detect was low (0.568) but slightly larger than TnT (0.550) in group 1. The *P*-values were not significant and the 95% CI included 0.5, which suggest that both tests were uninformative during this time period. The AUC for Cardio Detect^® ^remained lower than TnT in the remaining groups, a finding that was not in keeping with previous studies [33]. However, the AUC for Cardio Detect did increase over time before declining after the 24-h period. This could be explained by the release kinetics of circulating H-FABP as previously discussed.

The rationale of performing these two tests simultaneously is to exploit the advantages of the two cardiac biomarkers with different release kinetics. Combination of H-FABP, which is released early, and a later marker such as TnT may reduce the false-negative ratio and provide an optimal diagnostic performance [34]. Alpert et al. also suggested that different cardiac biomarkers, when performed simultaneously on patients with chest pain in the ED, may act synergistically and have a better diagnostic performance when used in combination than when interpreted individually.

This study demonstrated that the qualitative Cardio Detect^® ^test, which detects H-FABP in the circulation, was more sensitive than TnT and has a better NPV, especially during the early hours of AMI. Cardio Detect test may be potentially used to rule out myocardial infarction during the early phase of ischemic chest pain. However, there are still significant rates of false negatives even in the early hours of AMI, and further improvement should be made to the Cardio Detect^® ^test kit. This study also concluded that repeating the Cardio Detect^® ^test 1 h after an initial negative result improved the sensitivity, specificity, PPV, and NPV of the test, especially during the first 4 h after the onset of chest pain. The diagnostic accuracy of the repeat test was also superior to the Cardio Detect^® ^test alone or cardiac TnT during the early phase of chest pain. Therefore, if the initial Cardio Detect^® ^test is negative, a repeat test 1 h later is suggested, especially for patients who present early after the onset of chest pain.

This study agreed with previous recommendations that combination tests with different release kinetics (e.g., H-FABP and TnT) improved the diagnostic performance of cardiac biomarkers in detecting AMI, as compared to performing individual tests. It was shown that the combination test of Cardio Detect^® ^and TnT had a better diagnostic accuracy than an individual test, especially during the first 4 h after AMI. The combination test, however, may be redundant as TnT test alone was proven to be adequately sensitive and specific in diagnosing AMI, except for the early hours of chest pain. The Cardio Detect^® ^test was more sensitive in detecting AMI during the early hours of symptoms and has an added advantage of having a better NPV compared to TnT [35-37]. These characteristics of Cardio Detect^® ^are crucial since early exclusion of AMI depends on the sensitivity and NPV. A repeated Cardio Detect^® ^test an hour later is recommended if the initial test is negative, as this was proven to have better diagnostic indices. The combination test of Cardio Detect and TnT may be beneficial in selected patients, such as those who present with intermittent chest pain and are unsure or unable to recall the exact time of onset of chest pain. Combining the Cardio Detect^® ^and TnT would provide a wide safety net to diagnose AMI in these cases. With a high sensitivity and NPV, the combination test may be beneficial in ruling out myocardial infarction.

### Limitations

Several limitations were found during the study:

**1**. The Cardio Detect^® ^test kits were supplied in batches. Half way through the study, an updated version replaced the initial credit card-like test kit. The manufacturer reported that both test kits had similar characteristics, including the same cutoff point for a positive test to detect H-FABP. It is not known certainly whether the initial and updated versions of the Cardio Detect^® ^test kits were comparable in all aspects.

**2**. The attending medical officers may be biased when reading the Cardio Detect^® ^test result since they are not blinded to the history, physical examination, and ECG findings of the patient being investigated. Under ideal experimental conditions, the Cardio Detect^® ^test would have been read by a separate observer who is blinded to the patient's clinical condition. However, this was not possible in a busy emergency department setting.

**3**. The subjective nature of the reports of the patients about the exact onset of their ischemic symptoms may potentially overestimate or underestimate the duration of their ischemic symptoms. This may have influenced the grouping of patients according to the predetermined time frame and eventually affect the diagnostic indices of the group studied.

**4**. Inter-observer variability between two observers reading the Cardio Detect^® ^test was assessed in this study. Care was taken to perform the Cardio Detect^® ^test (and TnT) using standardized methods, and interpretation was done in a similar environment in the ED. The Cardio Detect^® ^test kit result has a tendency to change over time, and it was read at the 15-min mark. There were instances when the second reader read the test beyond 15 min. This delay may have contributed to the different interpretation of the test and affected the kappa analysis to assess agreement beyond chance between the two readers.

## Conclusion

The Cardio Detect^® ^test is more sensitive and has a better NPV than troponin T during the first 12 h of AMI. Repeating the Cardio Detect^® ^test 1 h after an initial negative result does improve the diagnostic indices, especially during the first 4 h after the onset of chest pain. However, those who present with intermittent chest pain and are unsure or unable to recall the exact time of onset of chest pain may benefit from the combination test.

## Competing interests

The authors declare that they have no competing interests.

## Authors' contributions

NH planned the study methodology, processed the grant application, participated in data collection, and prepared the publication material. AS was responsible for literature review, data collection, statistical analysis, and patient sampling.
